# End of Volume XVIII

**Published:** 1903-06

**Authors:** 


END OF VOLUME XVIII. This issue completes the eighteenth year of the "Red Back," and it has been a success from the beginning. I start on the nineteenth round with renewed courage and a determination to *hold the lead,* and to lay over anything in the journal line in the Southwest. Advertisers attest their appreciation of its value as a "fetching" medium, by a continuous patronage of eighteen years. Eighteen down, and I've got the deadwood on the balance! Set 'em up again!

